# Insertion and deletion polymorphisms of the ancient *AluS* family in the human genome

**DOI:** 10.1186/s13100-017-0089-9

**Published:** 2017-04-24

**Authors:** Maria S. Kryatova, Jared P. Steranka, Kathleen H. Burns, Lindsay M. Payer

**Affiliations:** 1 Department of Pathology, Johns Hopkins University School of Medicine, Miller Research Building (MRB) Room 447, 733 North Broadway, Baltimore, MD 21205 USA; 2 McKusick-Nathans Institute of Genetic Medicine, Johns Hopkins University School of Medicine, Miller Research Building (MRB) Room 447, 733 North Broadway, Baltimore, MD 21205 USA

**Keywords:** Retrotransposon, Mobile element, SINE, *Alu*, *AluS*, Polymorphism, Structural variation, Mobilome

## Abstract

**Background:**

Polymorphic *Alu* elements account for 17% of structural variants in the human genome. The majority of these belong to the youngest *AluY* subfamilies, and most structural variant discovery efforts have focused on identifying *Alu* polymorphisms from these currently retrotranspositionally active subfamilies. In this report we analyze polymorphisms from the evolutionarily older *AluS* subfamily, whose peak activity was tens of millions of years ago. We annotate the *AluS* polymorphisms, assess their likely mechanism of origin, and evaluate their contribution to structural variation in the human genome.

**Results:**

Of 52 previously reported polymorphic *AluS* elements ascertained for this study, 48 were confirmed to belong to the *AluS* subfamily using high stringency subfamily classification criteria. Of these, the majority (77%, 37/48) appear to be deletion polymorphisms. Two polymorphic *AluS* elements (4%) have features of non-classical *Alu* insertions and one polymorphic *AluS* element (2%) likely inserted by a mechanism involving internal priming. Seven *AluS* polymorphisms (15%) appear to have arisen by the classical target-primed reverse transcription (TPRT) retrotransposition mechanism. These seven TPRT products are 3′ intact with 3′ poly-A tails, and are flanked by target site duplications; L1 ORF2p endonuclease cleavage sites were also observed, providing additional evidence that these are L1 ORF2p endonuclease-mediated TPRT insertions. Further sequence analysis showed strong conservation of both the RNA polymerase III promoter and SRP9/14 binding sites, important for mediating transcription and interaction with retrotransposition machinery, respectively. This conservation of functional features implies that some of these are fairly recent insertions since they have not diverged significantly from their respective retrotranspositionally competent source elements.

**Conclusions:**

Of the polymorphic *AluS* elements evaluated in this report, 15% (7/48) have features consistent with TPRT-mediated insertion, thus suggesting that some *AluS* elements have been more active recently than previously thought, or that fixation of *AluS* insertion alleles remains incomplete. These data expand the potential significance of polymorphic *AluS* elements in contributing to structural variation in the human genome. Future discovery efforts focusing on polymorphic *AluS* elements are likely to identify more such polymorphisms, and approaches tailored to identify deletion alleles may be warranted.

**Electronic supplementary material:**

The online version of this article (doi:10.1186/s13100-017-0089-9) contains supplementary material, which is available to authorized users.

## Background

While we have long appreciated differences between individual genomes, it is only recently that robust sequencing efforts have allowed us to begin to build a comprehensive catalog of human structural variants [1, 2]. Mobile element insertions are an important source of structural variation in the human genome, with *Alu* elements specifically accounting for 17% of structural variants [2, 3]. *Alu* elements are non-autonomous retrotransposons, relying on the protein machinery of Long INterspersed Element-1 (LINE-1, L1) for their propagation [4]. Classically, new *Alu* insertions occur by target-primed reverse transcription (TPRT). This mechanism of insertion requires the L1 encoded protein ORF2p, which contains an endonuclease domain and reverse transcriptase domain [4–6]. L1 ORF2p endonuclease has a preference to cleave the negative strand at 5′ TTTT/AA 3′ sites, but is capable of targeting a range of sequences [7–10]. The T-rich sequence on the cleaved negative strand then primes with the poly-A tail of the *Alu* transcript, allowing reverse transcriptase to synthesize a copy of the *Alu* [3]; premature termination of reverse transcription results in the integration of a 5′ truncated element. Because the positive strand is nicked downstream of the initial cleavage site, the newly integrated *Alu* element is flanked by direct repeats, resulting from a duplication of the sequence at the insertion site when the staggered break is repaired [3]. Thus, an *Alu* insertion having arisen by TPRT exhibits the following defining features [11]: (1) an intact 3′ end, (2) a 3′ poly-A tail, and (3) flanking target site duplications (TSDs).

Only a small subset of the 1.1 million *Alu* insertions in the human genome are capable of retrotransposition, and recent retrotransposition events have created thousands of polymorphic insertions [1, 3, 11–14]. Polymorphic *Alu* elements almost exclusively belong to the youngest *AluY* subfamilies [2, 3, 7, 11, 14, 15]. While there have been reports of polymorphic elements from the evolutionarily older *AluS* subfamily in humans [2, 13, 15, 16], polymorphic *AluS* insertions are generally not considered to be an important contributor to structural variation and most structural variant discovery efforts have not specifically focused on identifying these elements. In this report we present examples of polymorphic *AluS* elements, provide annotations of the sequences, and consider the mechanisms that likely created the polymorphisms. Thus, our work expands the potential significance of *AluS* elements in contributing to structural variation in the human genome and emphasizes the importance of identifying additional *AluS* polymorphisms.

## Results

### Identification of polymorphic *AluS* elements in the human genome

How retrotransposon variants in the human genome affect gene expression or phenotype remains poorly elucidated. To better understand the functional effects of these elements, we focus on polymorphic elements near loci associated with disease risk and pathogenesis [17]. We compiled a catalog of previously reported polymorphic *Alu* elements (see Methods) and from this list selected 112 *Alu* variants that map near genome-wide association study (GWAS) signals to Sanger sequence and fully annotate [17]. As expected, most (96%), are from the youngest *Alu* subfamilies, 46% *AluYa5* and 23% *AluYb8*, the most recently retrotranspositionally active subfamilies whose members account for the overwhelming majority of previously reported polymorphic *Alu* insertions [3]. Intriguingly though, 4% (*n* = 4) belong to the evolutionarily older *AluS* subfamily, which was most active 35–60 million years ago [18, 19] and is considered to have limited in vivo retrotransposition capability in humans in the modern era [7]. These results suggest that polymorphic *AluS* elements may contribute to structural variation in the human genome more than previously thought.

Structural variants involving *Alu* elements may either be deletion or insertion polymorphisms. Since the *AluS* subfamily is considered to have been largely inactive for tens of millions of years [18, 19], we expected that some portion of *AluS* polymorphisms would reflect deletion polymorphisms, arising when *AluS* elements are (imperfectly) excised by an interstitial deletion. On the other hand, *Alu* insertion polymorphisms classically arise by TPRT. Ongoing retrotransposition has resulted in thousands of *Alu* insertion polymorphisms in the human genome, mostly confined to the *AluY* subfamilies [3]. Remarkably, we found that two of the four polymorphic *AluS* elements near GWAS signals described above are full-length elements that have all three defining features of a TPRT-mediated insertion [11]: (1) an intact 3′ end, (2) a 3′ poly-A tail, and (3) flanking TSDs. Therefore, these *AluS* polymorphisms appear to have arisen by TPRT.

To further expand the list of polymorphic *AluS* elements, we considered data from the most comprehensive effort to characterize structural variation in humans – the 1000 Genomes Project. In the most recent analysis there were 49 polymorphic *AluS* elements reported [2]. This list includes one of the *AluS* elements discussed above, thus bringing the total to 52 polymorphic *AluS* elements to characterize in more detail.

### Confirming *AluS* subfamily classification

We first set out to confirm the *AluS* subfamily assignment of these 52 polymorphic elements using high stringency criteria (see Methods). Subfamily classification was performed using multiple established methods whenever possible.

**Fig. 1:**
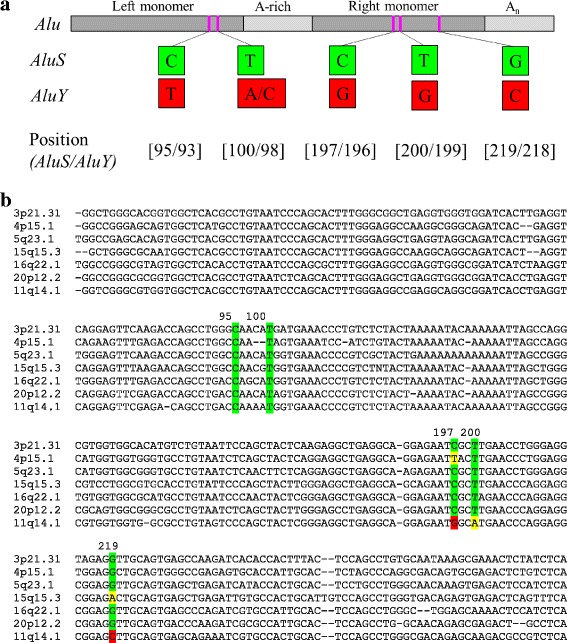
Diagnostic nucleotides differentiate *AluS* and *AluY* elements. **a** Five diagnostic nucleotides that distinguish RepBase consensus sequences of all six *AluS* subfamilies (*AluSc, AluSg, AluSp, AluSq, AluSx, AluSz,*) from the most common *AluY* subfamilies (*AluY, AluYa5, AluYa8, AluYb8, AluYb9, AluYc1*) were identified [3]. **b** Five diagnostic positions indicated in part (a) in the context of *Alu* sequence confirms *AluS* subfamily classification. The seven *AluS* TPRT insertion candidates are shown. *AluS* specific nucleotides at the diagnostic positions are highlighted in *green*, *AluY* specific nucleotides at the diagnostic positions are highlighted in *red*, and nucleotides at the diagnostic positions that are neither *AluS* nor *AluY* specific are highlighted in *yellow*. Further analysis of the polymorphic *Alu* element at 11q14.1, which has features of both *AluS* and *AluY* elements, that led to its ultimate classification as an *AluS* element is shown in Additional file 2: Figure S1

For the 49 *AluS* polymorphisms that are annotated in the reference genome (hg19), we compared the subfamily calls made by RepeatMasker [20], the RepeatMasker track of the UCSC Genome Browser (hg19), and the 1000 Genomes Project [2], (Additional file 1: Table S1). In 22 cases there was complete agreement among these sources. In 22 cases there was minor disagreement, limited to a discrepancy in classification among *AluS* subfamilies. In the remaining five cases there was a more substantial disagreement regarding subfamily classification. In the first of the five cases, there was a discrepancy between subfamily classification by the 1000 Genomes Project as an *AluSz* element and subfamily classification by both RepeatMasker and the RepeatMasker track of the UCSC Genome Browser (hg19) as an *AluJb* element; this element was ultimately classified as an *AluJ* element and excluded from later analysis. In the other four cases there was disagreement regarding the classification of the element in question as *AluS* versus *AluY*. To resolve this issue, we identified five diagnostic nucleotides that definitively distinguish between *AluS* and *AluY* consensus sequences [21] when considering the six *AluS* subfamilies and the six most common *AluY* subfamilies (Fig. 1a), [3]. The sequences of the four polymorphic *Alu* elements were manually evaluated with respect to these positions. Three elements were severely 5′ truncated making subfamily classification difficult. In particular, two elements were so short that they did not contain the necessary diagnostic positions to determine *AluY* versus *AluS* assignment, thus explaining the disagreement among the methods described above. Therefore, while these elements do have characteristics of classical TPRT insertions and may belong to the *AluS* subfamily, they were not included in our remaining analysis as they could not be confirmed to be *AluS* elements. The other element was less severely truncated than the two described above. It contained one diagnostic position distinguishing between *AluS* and *AluY* consensus sequences, which matched that of an *AluY* element, and was therefore also excluded from further analysis. The final element in question (located at 11q14.1) was full-length, which allowed for the evaluation of all five diagnostic positions between *AluS* and *AluY* consensus sequences illustrated in Fig. 1a. The polymorphic sequence matched the *AluS* consensus sequence at the two diagnostic positions in the left monomer, the *AluY* consensus sequence at two of the three diagnostic positions in the right monomer, and neither consensus sequence at the remaining nucleotide (Fig. 1b, Additional file 2: Figure S1). While such a chimeric element may have arisen by recombination between adjacent *AluS* and *AluY* elements [3], the fact that this polymorphic element is flanked by identical TSDs makes this possibility unlikely. Given the full-length nature of this *Alu* polymorphism, we considered seven additional positions that largely, although not definitively, distinguish *AluY* and *AluS* elements (Additional file 2: Figure S1). When considering all twelve positions, this element is consistent with only an *AluS* subfamily consensus sequence at six positions (highlighted in green) and consistent with only an *AluY* subfamily consensus sequence at three positions (highlighted in red). At one position (highlighted in gray) this element is consistent with both *AluS* and *AluY* subfamily consensus sequences and at two positions (highlighted in yellow) it is consistent with neither *AluS* nor *AluY* subfamily consensus sequences; evaluation at these positions was, thus, uninformative. Due to predominating *AluS* features, the polymorphic *Alu* element at 11q14.1 was ultimately classified as an *AluS* element and included in subsequent analysis.

Confirmation of subfamily classification of the three *AluS* polymorphisms not annotated in the reference genome was handled slightly differently. For two such *AluS* polymorphisms no subfamily assignment was made in the original report of the polymorphism; the element was only classified as belonging to the *Alu* family [14, 22] and subfamily assignment (to an *AluS* subfamily in both cases) was thus solely made using RepeatMasker [20]. For the third element, there was agreement between the original report [13] and RepeatMasker [20] with respect to subfamily assignment to the *AluSg* subfamily (Additional file 1: Table S1).

Further analysis thus focused on the 48 *Alu* polymorphisms confirmed to be *AluS* elements using these high stringency criteria.

### *AluS* deletion polymorphism candidates and Non-classical *Alu* insertion candidates

The overwhelming majority of previously reported polymorphic *AluS* elements in humans were classified as deletion polymorphisms [2, 13]. However, based on our analysis of the *AluS* variants mapping near GWAS signals, it appears that some extant *AluS* polymorphisms have features of a TPRT-mediated *Alu* insertion event. Therefore, we set out to categorize the 48 polymorphic *AluS* elements as insertion or deletion polymorphisms.

**Fig. 2:**
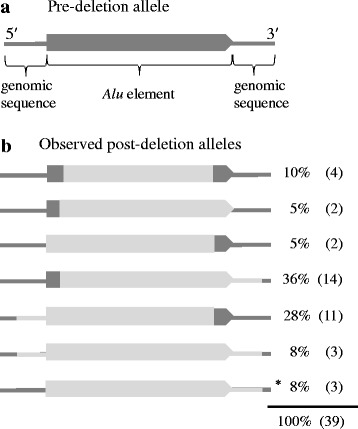
Characterization of the 39 *AluS* deletion polymorphism candidates. **a** The pre-deletion allele contains the *Alu* element (*dark gray block arrow*); flanking genomic sequence is depicted as a thin *dark gray* line on both sides of the *Alu* element. **b** Schematic of the seven categories of post-deletion alleles observed among the 39 *AluS* deletion polymorphism candidates. Polymorphic sequences are depicted in *light gray*; the *dark gray* parts indicate the sequences that do not vary among individuals. Deletion polymorphism candidates are defined as polymorphisms that encompass only a portion of the *Alu* element at that locus (e.g. the top five post-deletion allele categories) or that are not limited to only the *Alu* element (e.g. the bottom four categories). The last category (marked by an *asterisk*) includes two elements that have features of non-classical *Alu* insertions and may not be true deletion polymorphisms (see Results). All *Alu* elements are shown 5′ to 3′. Observed frequencies of each post-deletion allele category among the 39 *AluS* deletion polymorphism candidates are shown

Deletion polymorphisms may arise when fixed *Alu* elements are deleted through recombination, thus becoming polymorphic in the population. Such *Alu* deletions are often imprecise. While the pre-deletion allele contains the *Alu* element, the post-deletion allele lacks the *Alu* element, either in part or in its entirety, along with adjacent genomic sequence as well in some cases, depending on the recombination or end-joining event (Fig. 2). Thus, we defined deletion polymorphism candidates as polymorphisms that are not limited to the *Alu* element (i.e., due to the inclusion of adjacent genomic sequence) or that contain only a portion of the *Alu* element at that locus (i.e., only part of the *Alu* is variably present among individuals and the rest of the *Alu* is fixed). Of the 48 polymorphic *AluS* elements, 39 were initially identified to be deletion polymorphism candidates based on this definition (Fig. 2).

We considered the possibility that some of these 39 deletion polymorphism candidates we identified may instead reflect non-classical *Alu* insertions (NCAI). While we were confident that the 33 cases in which the polymorphism does not include the entire *Alu* element at that locus, so that part of the *Alu* is polymorphic and part is fixed (top five post-deletion allele categories in Fig. 2b), represent deletion polymorphisms, the six cases in which the polymorphism includes the entire *Alu* element as well as flanking genomic sequence (bottom two post-deletion allele categories in Fig. 2b) were evaluated more closely to determine if these could represent NCAI. These six polymorphisms could potentially be NCAI because over half of previously reported NCAI had 2 bp to 2 kb of non-*Alu* sequence inserted along with the *Alu* fragment [23].

NCAI have several other characteristic features that we considered in evaluating these six polymorphisms. NCAI are typically 3′ truncated, thus lacking a poly-A tail, and also lack flanking TSDs [23]. Because they arise by an endonuclease-independent (ENi) mechanism, no L1 ORF2p cleavage sites are observed at the insertion site [23]. Previous studies found that most NCAI are also associated with deletions at the insertion site ranging from 1 bp to ~7 kb [11, 23–25].

**Fig. 3:**
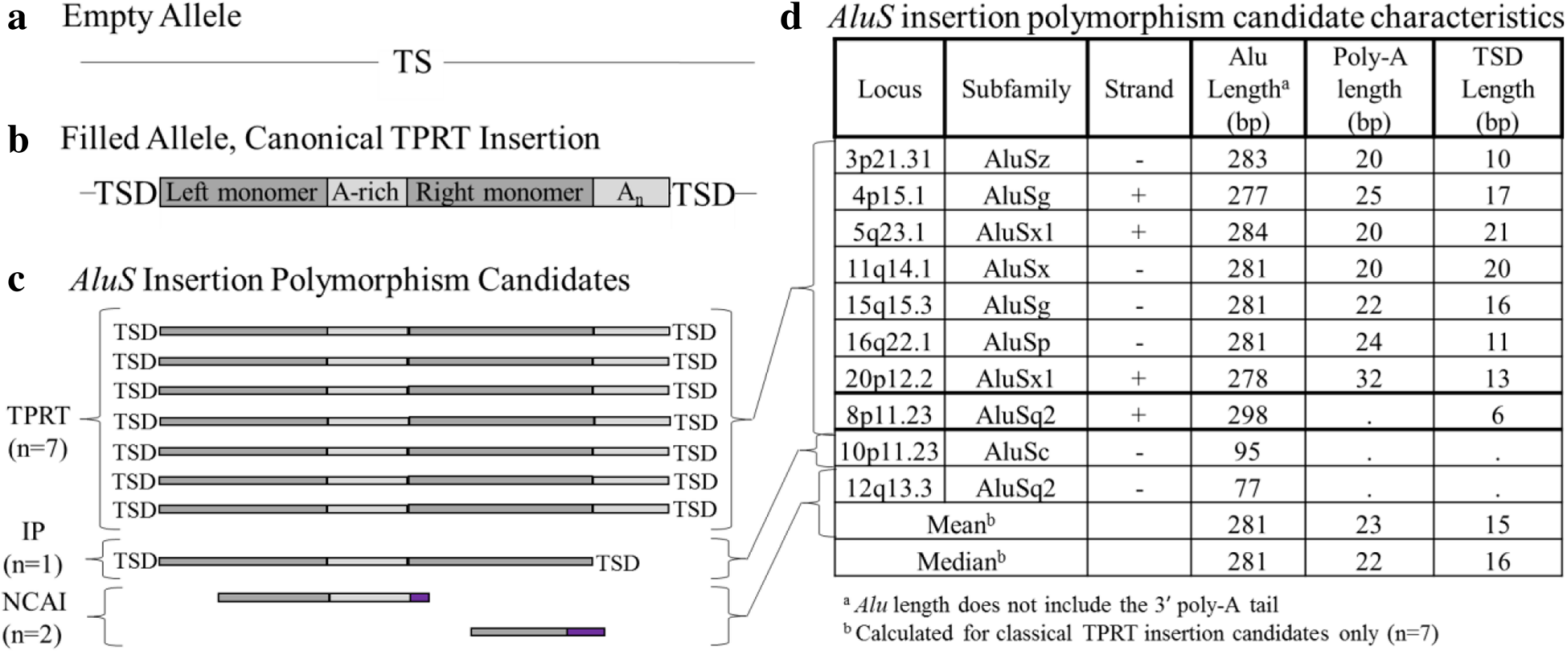
*AluS* insertion polymorphism candidates. **a** Empty (pre-insertion) allele prior to *AluS* element insertion with the target site (TS) sequence noted. **b** Filled allele after a classical TPRT insertion. The ~300 bp long *Alu* element consists of two monomers separated by an A-rich region, and also contains a 3′ poly-A tail (An). The TS sequence is duplicated (TSD) and flanks the *Alu* insertion. **c** Of the 11 initial *AluS* insertion polymorphism candidates, ten were PCR validated to be polymorphic in the population. Of these, seven (70%) are full-length elements with 3′ poly-A tails, flanked by TSDs, and are thus classical TPRT insertion candidates. One *AluS* insertion polymorphism candidate (10%) is full-length and flanked by TSDs, but lacks a 3′ poly-A tail, and thus likely arose by a mechanism involving internal priming (IP). Two *AluS* insertion polymorphism candidates (20%) are both 5′ and 3′ truncated, lack flanking TSDs, and include non-*Alu* sequence (shown in *purple*), thus exhibiting features of non-classical *Alu* insertions (NCAI). **d** Characteristics of *AluS* insertion polymorphism candidates

The three polymorphisms that include both 5′ and 3′ flanking genomic sequence in addition to the *AluS* element were all confirmed to be deletion polymorphisms. All three *AluS* elements included in these polymorphisms are full-length with 3′ poly-A tails, and are flanked by identical TSDs ranging from 11 to 17 bp; these features exclude the possibility that these are NCAI [23]. We next considered the three polymorphisms that include 3′ flanking genomic sequence in addition to the *AluS* element in its entirety. One of these was confirmed to be a deletion polymorphism, by virtue of being a full-length *AluSz* element with a 3′ poly-A tail; flanking TSDs could not be identified due to the presence of another *Alu* insertion immediately 5′ of this element. The two remaining polymorphisms in this category appear to be NCAI (Fig. 3, Additional file 3: Table S2). Both of these elements are *Alu* fragments, truncated at both the 5′ and 3′ ends, one with 95 bp of homology to the left monomer and A-rich region of the *AluSc* consensus sequence and the other with 77 bp of homology to the right monomer of the *AluSq2* consensus sequence. Flanking TSDs are not observed in either case. No previously reported L1 ORF2p endonuclease cleavage sites [7] are present at either insertion site, consistent with ENi insertion [23]. Both polymorphisms include short stretches of non-*Alu* sequence at the 3′ end (11 bp with the *AluSc* fragment and 21 bp with the *AluSq2* fragment); the polymorphism that includes the *AluSq2* fragment is also associated with a 14 bp deletion at the insertion site. These features are thus consistent with those previously reported for NCAI [23]. Both of these NCAI candidates were PCR validated to be polymorphic in the population (Additional file 3: Table S2).

In summary, of the 48 polymorphic *AluS* elements, 77% (37/48) are deletion polymorphism candidates (Additional file 4: Table S3) and 4% (2/48) appear to be NCAI. The remaining nine insertion polymorphism candidates were next evaluated in more detail.

### Polymorphic *AluS* insertion likely arising by internal priming

One *AluS* insertion polymorphism candidate, which was PCR validated to be polymorphic in the population (Additional file 3: Table S2), does not have all of the defining characteristics of retrotransposon insertions occurring by TPRT [11]. The polymorphic *AluSq2* element at 8p11.23 is full-length and flanked by TSDs but completely lacks a 3′ poly-A tail (Fig. 3, Additional file 3: Table S2). Thus, it likely arose through an insertion mechanism other than TPRT. Specifically, its features are characteristic of an element that inserted by internal priming [26]. While in classic TPRT, reverse transcription begins at the 3′ end of the poly-A tail [27], in this case reverse transcription likely began at the 5′ end of the poly-A tail, thus accounting for the insertion of a full-length element, only lacking the poly-A tail. While poly-A tail length tends to decrease over time after insertion toward a more stable equilibrium value, the poly-A tail is unlikely to be completely eliminated and no such cases have been reported even among older *Alu* subfamilies [27]. Thus, insertion of this tail-less polymorphic *AluSq2* element most likely occurred by internal priming. Absence of an L1 ORF2p endonuclease cleavage site at the insertion site of this element is consistent with the fact that the internal priming mechanism of insertion does not always rely on L1 ORF2p endonuclease and may occur at the site of staggered double-strand breaks (DSBs), thus creating flanking TSDs [26].

### Polymorphic *AluS* insertions likely arising by target-primed reverse transcription

Eight *AluS* polymorphisms have all the defining features of retrotransposon insertions that have arisen by TPRT [11]). However, we were only able to PCR validate seven of them to be polymorphic in the population (Additional file 3: Table S2). Therefore, further analysis only focused on the seven validated *AluS* TPRT insertion candidates (Figs. 1b and 3).

**Fig. 4:**
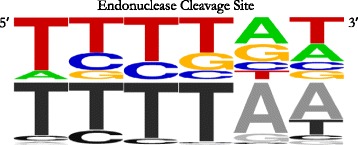
L1 ORF2p endonuclease cleavage sites for all classical TPRT insertion candidates (*n* = 7) displayed as a WebLogo diagram [47]. The negative strand is depicted, 5′ to 3′. The published consensus sequence is depicted below in grayscale [7]

These seven *AluS* elements are full-length insertions (277–284 bp), with intact 3′ poly-A tails (20–32 bp), and are flanked by TSDs (10–21 bp) (Fig. 3, Additional file 3: Table S2). To evaluate further the possibility of TPRT-mediated insertion, we searched for the L1 ORF2p endonuclease cleavage site at the insertion site of each of the seven elements (Fig. 4, Additional file 3: Table S2). The endonuclease cleavage sites for the seven loci fall within the distribution previously reported by Konkel et al. [7] (Fig. 4, Additional file 3: Table S2). Thus, we see features consistent with TPRT-mediated insertion in seven polymorphic *AluS* elements of 48 total *AluS* polymorphisms evaluated in this report (15%).

### Percent divergence from subfamily consensus sequence and estimated Age of TPRT insertion candidates

These seven *AluS* insertion candidates could reflect fairly recent TPRT-mediated insertions, or could be old insertions slow to reach fixation in human populations. To consider the age of these sequences relative to other *AluS* elements, we compared them to their respective subfamily consensus sequences. Relatively new insertions would not have had much time to accumulate random mutations (i.e., neutral substitutions) and would conserve many of the features of *Alu* elements required for retrotransposition.

**Fig. 5:**
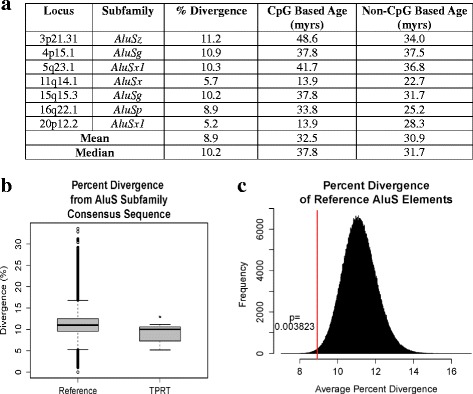
Estimated age and degree of divergence from subfamily consensus sequence of *AluS* TPRT insertion candidates. **a** For each of the *AluS* TPRT insertion candidates (*n* = 7) percent divergence from the respective *AluS* subfamily consensus sequence is shown along with estimated ages for the elements based on CpG and non-CpG substitution rates. **b** Boxplot of percent divergence from the respective *AluS* subfamily consensus sequence of all *AluS* elements annotated in the reference genome (*n* = 686,955) and the TPRT insertion candidates (*n* = 7). **c** The TPRT insertion candidates (*n* = 7) are significantly less diverged from their respective *AluS* subfamily consensus sequence than are all the *AluS* elements annotated in the reference genome (*n* = 686,955) from their respective subfamily consensus sequence (*p* = 0.0038, permutation test). The distribution of the mean percent divergence of 1 × 10^6^ random samples of *n* = 7 drawn from the total 686,955 *AluS* elements annotated in the reference genome is shown. The mean percent divergence of the seven TPRT insertion candidates is shown as a *vertical red line*

We evaluated the degree of divergence of each *AluS* TPRT insertion candidate from its subfamily consensus sequence, and found that the divergence ranges from 5.2 to 11.2%, with a mean of 8.9% and a median of 10.2% (Fig. 5a). We found that these seven TPRT insertion candidates are significantly less diverged from their respective subfamily consensus sequence than are all the *AluS* elements annotated in the reference genome (*n* = 686,955) from their respective subfamily consensus sequence (*p* = 0.0038, permutation test), (Fig. 5b-c). This supports the hypothesis that these insertions likely occurred more recently than at the peak *AluS* activity approximately 35–60 million years ago [18, 19].

We also estimated the age of the TPRT insertion candidates using previously reported substitution rates at CpG and non CpG sites. Since *Alu* elements are rich in CpG sites, which are known to have an appreciably higher mutation rate than non-CpG sites [28], CpG and non-CpG based age estimates are both valuable metrics in addition to percent divergence from the consensus sequence. Age estimates were calculated for each TPRT insertion candidate as previously described [28–32] (see Methods). CpG based age estimates range from 13.9 to 48.6 million years and non-CpG based age estimates range from 22.7 to 37.5 million years (Fig. 5a). Despite the range of estimated ages among the TPRT insertion candidates, overall they appear to have inserted more recently than at the peak of *AluS* activity 35–60 million years ago [18, 19].

### Conservation of functionally significant *Alu* sequence features in TPRT insertion candidates

To evaluate the degree of conservation of functionally significant *Alu* sequence features, we focused on three specific regions – the RNA polymerase III promoter A and B boxes within the left monomer [33], the SRP9/14 major and minor binding sites within the left and right monomers [16], and AC dinucleotides within the left and right monomers previously reported to be important for maintaining *Alu* RNA secondary structure [34]. Since these sequences are critical for successful retrotransposition, they would presumably be present and functional in the source element templating each TPRT insertion variant, and would be highly conserved in recent insertions.

**Fig. 6:**
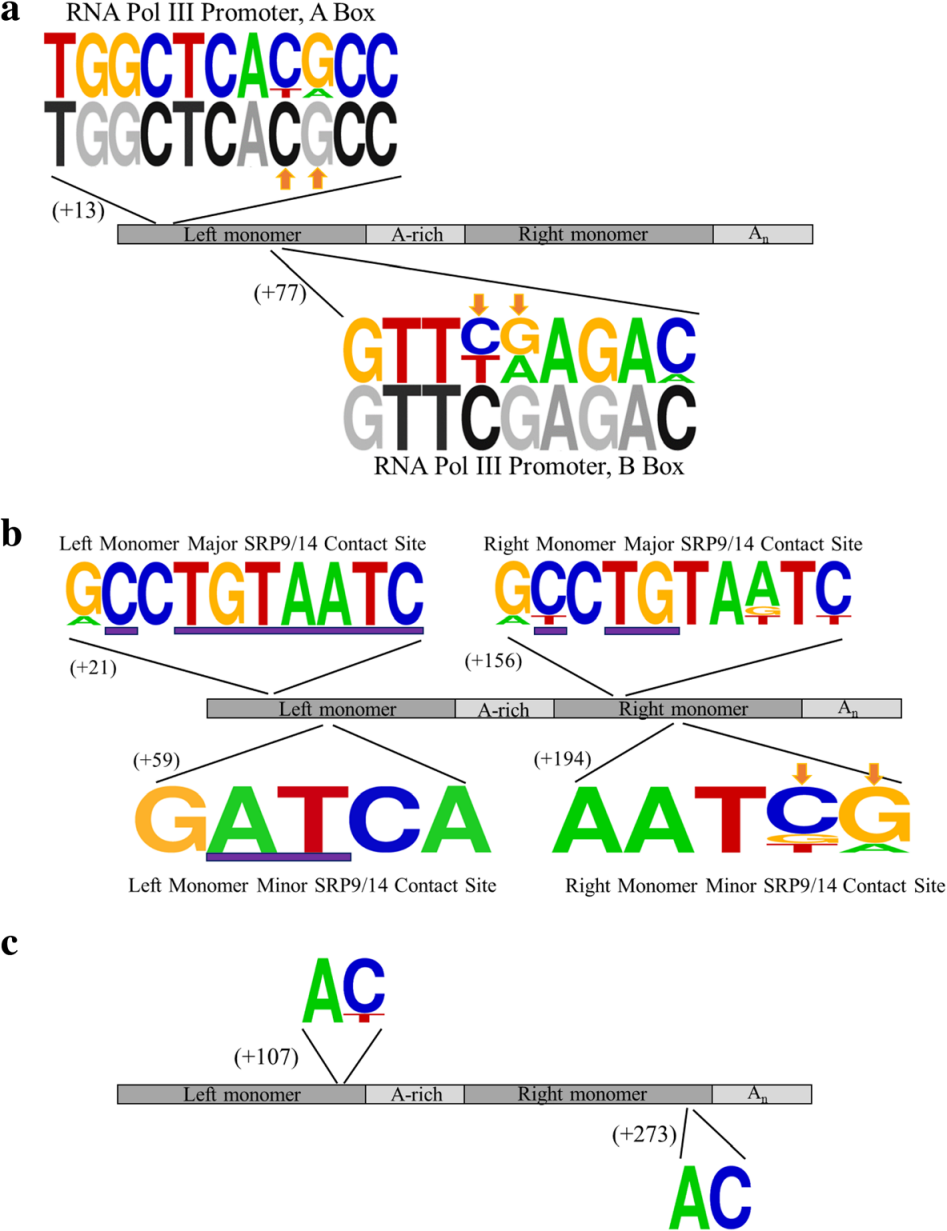
Conservation of functionally significant *Alu* sequence features in the seven classical TPRT insertion candidates. **a** WebLogo diagram [47] of the RNA polymerase III promoter A and B boxes, with the published consensus sequences depicted below in grayscale [33]. CpG sites are indicated by an *orange* arrow. **b** WebLogo diagram [47] of the SRP9/14 binding sites. Previously reported most highly conserved sites within the SRP9/14 binding sites of elements capable of retrotransposition are underlined by a *purple* bar [16]. CpG sites are indicated by an *orange* arrow. **c** WebLogo diagram [47] of two AC dinucleotides in the *Alu* sequence reported to play a critical role in maintaining the closed loop conformation of *Alu* RNA that is important for interaction with SRP9/14 and efficient retrotransposition [34]

The RNA polymerase III promoter, which is important for efficient transcription [33], is indeed well conserved (Fig. 6a, Additional file 5: Figure S2). Nine of the 11 nucleotides in the A box consensus sequence are fully conserved in all seven *AluS* elements, with infrequent departures from the consensus at the other two positions; five *AluS* elements have an A box that exactly matches the consensus sequence, and the remaining two elements have one mismatch, each at a different position. Six of the nine nucleotides in the B box consensus sequence are fully conserved in all seven *AluS* elements, with some variation at the other three positions; two *AluS* elements have a B box that exactly matches the consensus sequence, four elements have one mismatch, and one element has three mismatches. Two *AluS* elements have both an A and a B box that exactly matches the consensus sequence. Across the A and B boxes, four of the five imperfectly conserved positions are at CpG sites, which are known to have a higher mutation rate due to spontaneous deamination of methylated cytosines at these positions [28].

The SRP9/14 binding sites, which, as predicted by the ribosome-binding model, are important for interaction with SRP9/14, and subsequently with the ribosome and L1 retrotransposition machinery [16, 35] are also highly conserved (Fig. 6b, Additional file 5: Figure S2). In the left monomer, all of the previously reported most highly conserved nucleotides in the major and minor binding sites of elements capable of retrotransposition were fully conserved in all seven *AluS* elements. In the right monomer, two of the three nucleotides previously reported to be most highly conserved in the major binding site of elements capable of retrotransposition were fully conserved in all seven *AluS* elements, and the third nucleotide was conserved in six of the seven *AluS* elements. In all, six of the seven *AluS* elements have full conservation of all the nucleotides previously reported to be most highly conserved within the SRP9/14 binding sites of elements capable of retrotransposition.

Finally, there was strong conservation of both the left and right monomer AC dinucleotides (Fig. 6c, Additional file 5: Figure S2), [34]. These positions have been reported to play a critical role in stabilizing the closed loop conformation of *Alu* RNA that is important for interaction with SRP9/14 via the SRP9/14 binding sites [34] thus lending further support to the ribosome-binding model [35]. The left monomer AC dinucleotide was largely conserved among the seven *AluS* TPRT insertion candidates; there was no variation at the A position, and variation at the C position in only one of the seven *AluS* TPRT insertion candidates. There was perfect conservation of the right monomer AC dinucleotide in all seven *AluS* elements.

## Discussion

In this report, we annotate 52 previously reported polymorphic *AluS* elements, confirm that 48 of them do indeed belong to the *AluS* subfamily using high stringency criteria, and comment on their likely mechanism of origin. While most of these appear to be deletion polymorphisms consistent with previous reports [2, 13], we present evidence that seven of these polymorphic *AluS* elements have features consistent with insertion by TPRT. This implies that some *AluS* elements may have been more active recently than previously thought [7, 19] and expands the significance of *AluS* retrotransposition in contributing to structural variation in the human genome.

The overwhelming majority of *AluS* insertions in the human genome are fixed, consistent with the fact that the *AluS* subfamily was most active 35–60 million years ago [18, 19]. Based on this, most *AluS* elements that are polymorphic among humans are expected to be deletion polymorphisms. Deletion events are highly unlikely to include only the *Alu* element in its entirety; while a recombination event between the flanking TSDs would yield a precise deletion of the intervening *Alu* element, such events are extremely rare [36]. Thus, we defined a deletion polymorphism candidate as one that was not limited to only the *Alu* element or did not contain the *Alu* element at that locus in its entirety. As expected, the majority (77%, 37/48) of polymorphic *AluS* elements evaluated in this study are deletion polymorphism candidates.

The remaining 23% (11/48) of *AluS* polymorphisms characterized in this report are insertion polymorphism candidates, of which ten were PCR validated to be polymorphic in the population. Three mechanisms of *Alu* insertion have been previously reported – TPRT [4], internal priming (IP) [26], and NCAI/ENi [23]– and are all represented among the ten *AluS* insertion polymorphism candidates (Fig. 3). Two insertion polymorphisms have features of NCAI [23]. The *AluS* elements included in these polymorphisms are both 5′ and 3′ truncated and lack flanking TSDs; no previously reported L1 ORF2p endonuclease cleavage sites could be identified at the insertion site. Both polymorphisms include non-*Alu* sequence at the 3′ end and one polymorphism is associated with a deletion at the insertion site. These are all characteristic features of ENi insertion, a mechanism in which *Alu* transcripts are utilized by the cell to repair DSBs, thus leading to insertion polymorphisms [23]. One *AluS* insertion polymorphism appears to have arisen by a mechanism involving IP [26]. While this element is full-length and flanked by TSDs, it lacks a 3′ poly-A tail and no L1 ORF2p endonuclease cleavage site could be identified at the insertion site. These features are consistent with insertion by IP, which may be an alternative mechanism to repair staggered DSBs [26].

The majority of *AluS* insertion polymorphism candidates (70%, 7/10) have all the features of classical TPRT insertions, which may suggest that *AluS* elements were more active recently than previously thought. Our observation is consistent with the “stealth driver” model, which posits that some subfamily members retain low levels of activity over tens of millions of years, long after the subfamily’s peak activity [37]. The seven TPRT insertion candidates do not appear to all be products of a single persistent “stealth-driver” element, however. These elements belong to four different subfamilies (three *AluSx* elements, two *AluSg* elements, one *AluSp* element, and one *AluSz* element), which strongly implies that multiple source elements contributed to these insertions.

The ability of *AluS* elements to retrotranspose in humans is considered minimal due to the fact that few polymorphic insertions with features consistent with TPRT-mediated insertion have been identified [7]. However, a de novo *AluSq/Sp* insertion in an exon of *BRCA1* with features of TPRT-mediated insertion has been reported, thus suggesting that some *AluS* elements may still be retrotransposition competent and may, moreover, influence disease risk [38]. Furthermore, *AluS* consensus sequences and some genomic *AluS* elements have been shown to be active in in vitro retrotransposition assays [16]. Importantly, those elements diverging more than 10% from their consensus sequence are inactive, thus highlighting the importance of *Alu* sequence integrity for retrotransposition capability [16]. If the seven candidate TPRT insertions we evaluated did indeed occur fairly recently, then the sequences would not have had much time to accumulate random mutations and would not yet significantly diverge from their respective subfamily consensus sequences. This is indeed what we observe, and the seven TPRT insertion candidates, with a mean percent divergence of 8.9%, are significantly less diverged from their respective subfamily consensus sequence than are all the *AluS* elements annotated in the reference genome from their respective subfamily consensus sequence (*p* = 0.0038, permutation test).

Similarly, specific sequence features known to be functionally important for retrotransposition, namely the RNA polymerase III promoter, SRP9/14 binding sites, and AC dinucleotides involved in maintaining *Alu* RNA secondary structure, are highly conserved. The RNA polymerase III promoter is important for efficient transcription of the element [33], and the AC dinucleotides play a critical role in maintaining the closed loop conformation of *Alu* RNA that is important for interaction with SRP9/14 via the SRP9/14 binding sites, which thus allows the *Alu* transcript to associate with the ribosome and ultimately positions it in close proximity to the L1 encoded proteins required for retrotransposition [16, 34, 35, 39, 40]. The high overall level of conservation suggests these seven TPRT candidates have not diverged significantly from elements that would be capable of retrotransposition, consistent with a recent insertion templated by a TPRT competent element.

While polymorphic *AluS* elements have previously been reported, most discovery efforts aimed to identify only young *Alu* subfamilies and did not include *AluS* subfamily consensus sequences in their algorithms [2, 12, 41]. The *AluS* polymorphisms reported by the 1000 Genomes Project (phase 3) [2] were identified using a pipeline for mapping insertion/deletion polymorphisms rather than the Mobile Element Locator Tool; thus, their identification of polymorphic *AluS* elements was limited to those that are in the reference genome. Notably, a recent study by Hormozdiari et al. [13] that did intend to identify polymorphic *AluS* elements, reports that 9.4% of identified polymorphic *Alu* elements belong to the *AluS* subfamily. However, the authors inferred that the majority of these were likely deletion polymorphisms or insertions arising by endonuclease-independent mechanisms as opposed to novel TPRT events [13]. Here, we present evidence that while, in agreement with previous studies, the majority (77%, 37/48) of the polymorphic *AluS* elements evaluated in this report are likely deletion polymorphisms, 15% (7/48) have features of TPRT-mediated insertions. While these may represent insertions that occurred at the peak of *AluS* activity and have by some means been slow to progress to fixation in the population, the strong conservation of sequence features important for retrotransposition (e.g. RNA polymerase III promoter, SRP9/14 binding sites) implies that some of these may be fairly recent insertions. As most previous discovery efforts did not specifically target identification of *AluS* polymorphisms [2, 12, 41], the majority of the polymorphic *AluS* TPRT candidates described here were identified because there were no reads mapping to the annotated *Alu* element in the reference genome. Therefore, there is a potential for more polymorphic *AluS* elements, especially less common variants not already annotated in the reference genome, to be identified with targeted discovery efforts.

## Conclusions

In summary, we present evidence of polymorphic *AluS* elements in the human genome with features consistent with TPRT-mediated insertion events. These findings imply that multiple *AluS* subfamilies may have been more active recently than previously thought, consistent with the “stealth driver” model [37]. Our analysis also substantiates the concept that *AluS* element deletions are an important contributor to structural variation in humans. In fact, since most methodologies for finding insertion variants have focused on identifying polymorphic *AluY* elements, we expect there to be more yet uncharacterized polymorphic *AluS* insertions and deletions in the human genome. Our findings stress the importance of future structural variant discovery efforts to identify polymorphic *AluS* elements.

## Methods

### Cataloging polymorphic *Alu* elements

We compiled a list of previously reported polymorphic *Alu* elements [12–14, 22, 41–44], totaling 13,572 *Alu* variants across the human genome. To focus on *Alu* variants with potential functional consequences, we selected polymorphic *Alu* elements within linkage disequilibrium (LD) blocks (*r* ^*2*^ ≥ 0.8) around GWAS trait-associated SNPs (TAS) with *p* < 10^−9^ from the NHGRI-EBI GWAS catalog [45] and Sanger sequenced 112 of them. We used RepeatMasker [20] to make subfamily assignments [17].

We also considered the list of polymorphic *AluS* elements reported by the 1000 Genomes Project, phase 3 [2]. In this report [2], the *Alu* polymorphisms of interest were identified using deletion discovery algorithms, and then classified as *AluS* using the AluScan algorithm as part of the Mobile Element Locator Tool (MELT).

### Classifying polymorphic *Alu* elements by subfamily

Subfamily assignment of the 52 *AluS* polymorphisms ascertained for this study was confirmed using high stringency criteria, using multiple established methods when possible. Subfamily assignments for all 52 elements were made using RepeatMasker [20].

For the 49 elements annotated in the reference genome, subfamily assignments from RepeatMasker [20], the UCSC Genome Browser RepeatMasker track (hg19), and the 1000 Genomes Project (obtained using the AluScan algorithm as part of the Mobile Element Locator Tool (MELT)) [2] were compared (Additional file 1: Table S1). When there was disagreement in classification among *AluS* subfamilies, the final assignment was made in accordance with RepeatMasker [20], both because it is a well trusted tool and for the sake of consistency, since it is the main tool available to classify the three non-reference genome polymorphic *AluS* elements presented in this report. When there was more substantial disagreement with respect to *AluS* versus *AluY* assignment, the elements were manually classified. RepBase consensus sequences [21] for the following subfamilies were obtained (*AluSc, AluSg, AluSp, AluSq, AluSx, AluSz, AluY, AluYa5, AluYa8, AluYb8, AluYb9, AluYc1*) and aligned using the MUSCLE multiple sequence alignment tool [46], (Additional file 2: Figure S1). Five diagnostic nucleotides that distinguish all six *AluS* subfamilies from the six *AluY* subfamilies included in the alignment were identified (Fig. 1a), [3]. The polymorphic *Alu* sequences were evaluated at these positions and the final assignment was made based on which consensus sequence the element more closely resembled.

For the three elements not annotated in the reference genome, the RepeatMasker [20] classification was compared to the subfamily classification indicated in the original report of the polymorphism, if available (Additional file 1: Table S1). When no subfamily classification was made in the original report, subfamily assignment was made solely using RepeatMasker.

### Annotating reference genome polymorphic *Alu* elements

Forty-five of the 48 polymorphic *Alu* elements confirmed to belong to the *AluS* subfamily are in the reference genome (hg19). To confirm the extent of the polymorphism, we compared the annotated polymorphic sequence [2] to the reference genome (hg19) and the RepeatMasker track (UCSC Genome Browser, hg19). Deletion polymorphisms were initially identified as those not limited to only the *Alu* element (i.e., due to the inclusion of adjacent genomic sequence) or those that did not contain the *Alu* element at that locus in its entirety (i.e., part of the *Alu* is polymorphic among individuals and the rest of the *Alu* is present in everyone). Deletion polymorphism candidates that included the entire *Alu* element as well as additional 5′ and/or 3′ flanking genomic sequence were further investigated to determine whether they could represent NCAI; this was done by evaluating the *Alu* element included in the polymorphism for degree of truncation, presence of a 3′ poly-A tail, and flanking TSDs. Further analysis focused only on the insertion polymorphism candidates. When present, the 3′ poly-A tail was identified. Flanking genomic sequence was obtained from the reference genome and used to manually identify TSDs; TSD length was maximized at the expense of poly-A tail length when applicable.

### Annotating non-reference genome polymorphic *Alu* elements

Three of the 48 polymorphic *Alu* elements confirmed to belong to the *AluS* subfamily are not in the reference genome (hg19). These loci were PCR amplified with primers flanking the insertion site from individuals from the Centre d’Etude du Polymorphisme Humain (CEPH) from Utah (CEU) HapMap Reference panel to obtain sequences for the filled and empty alleles with respect to each *Alu* element. These PCR products were then cloned and Sanger sequenced. The resulting sequences of the filled and empty alleles were aligned to each other and the reference genome (hg19) to determine the extent of the polymorphism, including the position of the breakpoints with respect to the *Alu* sequence. The identified polymorphic sequence was then analyzed by RepeatMasker [20] to determine which parts of the polymorphic sequence had *Alu* homology and make a subfamily assignment. When present, the 3′ poly-A tail and TSDs were manually identified and annotated as described in the section above.

### PCR validation of insertion polymorphism candidates

Primers flanking the insertion site of the 11 insertion polymorphism candidate elements were designed, the regions were PCR amplified, and the PCR amplicons were resolved using gel electrophoresis. Polymorphic *AluS* elements were confirmed by amplification of two different sized alleles from the locus, that, when viewed on the agarose gel, differ in size corresponding to the size of the respective *AluS* element. Loci at which two alleles, the pre-insertion allele and *AluS*-containing allele, could be observed were validated polymorphisms. Additional file 3: Table S2 includes the DNA samples and PCR primer sequences used for validation of each locus.

### Percent divergence from *AluS* subfamily consensus sequence

For the TPRT insertion candidates classified as *AluS* elements by RepeatMasker [20] (*n* = 6), the percent divergence from the respective subfamily consensus sequence was obtained from that analysis. For the *Alu* element at 11q14.1, the percent divergence from the RepBase *AluSx* consensus sequence [21] was determined using the MUSCLE multiple sequence alignment tool [46]. To extend analysis to all *AluS* elements the genome, we used the Table Browser function in the UCSC Genome Browser to obtain for all *AluS* elements annotated in the RepeatMasker track (hg19) the percent divergence from the respective *AluS* subfamily consensus sequence. A permutation test was performed using R version 3.2.3 to determine whether the mean percent divergence of the seven TPRT insertion candidates $\left(\overline{x}=8.914\right)$ was significantly lower than the mean percent divergence of all *AluS* elements in the reference genome. From the total 686,955 *AluS* elements annotated in the reference genome, 1 × 10^6^ random samples of *n* = 7 were drawn with replacement, and the mean percent divergence of each sample was calculated to obtain a distribution of the means (X), (Fig. 5c). The *p*-value (*p* = 0.003823) was calculated as the fraction of random samples with means less than the observed percent divergence of the seven TPRT insertion candidates $\left( \Pr \left( X<\overline{x}\right)\right)$.

### *Alu* element age estimates

Two estimates for the age of the *Alu* elements were calculated based on CpG and non-CpG substitution rates as previously reported [28–32]. Briefly, *AluS* TPRT insertion candidate sequences (without the poly-A tail) were aligned to the respective *AluS* subfamily RepBase consensus sequence [21] using the MUSCLE multiple sequence alignment tool [46]. The number of substitutions at CpG and non-CpG sites were counted; for the CpG sites, only C to T and G to A substitutions were counted. The substitution densities were then calculated by dividing the number of observed CpG (or non-CpG) substitutions by the total number of CpG (or non-CpG) sites in the consensus sequence. The age of these elements was then calculated using a neutral rate of evolution of k = 1.5 × 10^−9^ per nucleotide position per year for the non-CpG sites [31] and a six-fold higher rate of evolution (k = 9 × 10^−9^ per nucleotide position per year) for the CpG sites as determined by Xing et al. [28].

## Additional files

Additional file 1: Table S1. Comparison of subfamily classification of *Alu* polymorphisms (*n* = 52) using multiple established methods. (XLSX 14 kb)

Additional file 2: Figure S1. Diagnostic nucleotides differentiate *AluS* and *AluY* subfamily consensus sequences. **a**. To identify diagnostic nucleotides differentiating *AluS* and *AluY* subfamily consensus sequences, RepBase consensus sequences of all six *AluS* subfamilies (*AluSc, AluSg, AluSp, AluSq, AluSx, AluSz,*) and the most common *AluY* subfamilies (*AluY, AluYa5, AluYa8, AluYb8, AluYb9, AluYc1*) were aligned. Five diagnostic nucleotides that distinguish all six *AluS* subfamilies from six *AluY* subfamilies included in the alignment were identified (highlighted in magenta). Seven additional positions that largely, but not definitively, distinguish between *AluS* and *AluY* elements are also illustrated (highlighted in cyan). **b**. Full-length polymorphic *Alu* element at 11q14.1 that has features of both *AluS* and *AluY* elements. Manual evaluation at the 12 diagnostic nucleotides that differentiate *AluS* and *AluY* elements led to its final classification as an *AluS* element due to predominating *AluS* features. This element is consistent with only an *AluS* subfamily consensus sequence at six positions (highlighted in green) and consistent with only an *AluY* subfamily consensus sequence at three positions (highlighted in red). At one position (highlighted in gray) this element is consistent with both *AluS* and *AluY* subfamily consensus sequences and at two positions (highlighted in yellow) it is consistent with neither *AluS* nor *AluY* subfamily consensus sequences; evaluation at these positions was, thus, uninformative. (PDF 72 kb)

Additional file 3: Table S2. Polymorphic *AluS* insertion candidates (*n* = 11). (XLSX 17 kb)

Additional file 4: Table S3. Sequences of deletion and *Alu*-containing alleles of *AluS* deletion polymorphisms (*n* = 37) [2]. (XLSX 16 kb)

Additional file 5: Figure S2. Functionally significant *Alu* sequence features annotated in TPRT insertion candidates (*n* = 7). (PDF 203 kb)

## Abbreviations

CEPH: Centre d’Etude du Polymorphisme Humain
CEU: Centre d’Etude du Polymorphisme Humain (CEPH) from Utah
DSB: Double-strand break
ENi: Endonuclease-independent
GWAS: Genome-wide association study
IP: Internal priming
LD: Linkage disequilibrium
LINE-1, L1: Long INterspersed Element-1
MELT: Mobile Element Locator Tool
NCAI: Non-classical *Alu* insertion
NHGRI-EBI: National Human Genome Research Institute – European Bioinformatics Institute
ORF2p: Open reading frame 2 protein
SNP: Single nucleotide polymorphism
SRP: Signal recognition particle
TAS: Trait-associated SNP
TPRT: Target-primed reverse transcription
TSD: Target site duplication
