# Supplementary material for: Insertion and deletion polymorphisms of the ancient AluS family in the human genome
Source: Mob DNA. 2017 Apr 24;8:6. doi: 10.1186/s13100-017-0089-9 (PMC5402677; doi:10.1186/s13100-017-0089-9)
Supplement: Supplementary file 2 — Diagnostic nucleotides differentiate AluS and AluY subfamily consensus sequences. a. To identify diagnostic nucleotides differentiating AluS and AluY subfamily consensus sequences, RepBase consensus sequences of all six AluS subfamilies (AluSc, AluSg, AluSp, AluSq, AluSx, AluSz,) and the most common AluY subfamilies (AluY, AluYa5, AluYa8, AluYb8, AluYb9, AluYc1) were aligned. Five diagnostic nucleotides that distinguish all six AluS subfamilies from six AluY subfamilies included in the alignment were identified (highlighted in magenta). Seven additional positions that largely, but not definitively, distinguish between AluS and AluY elements are also illustrated (highlighted in cyan). b. Full-length polymorphic Alu element at 11q14.1 that has features of both AluS and AluY elements. Manual evaluation at the 12 diagnostic nucleotides that differentiate AluS and AluY elements led to its final classification as an AluS element due to predominating AluS features. This element is consistent with only an AluS subfamily consensus sequence at six positions (highlighted in green) and consistent with only an AluY subfamily consensus sequence at three positions (highlighted in red). At one position (highlighted in gray) this element is consistent with both AluS and AluY subfamily consensus sequences and at two positions (highlighted in yellow) it is consistent with neither AluS nor AluY subfamily consensus sequences; evaluation at these positions was, thus, uninformative. (PDF 72 kb) [file 13100_2017_89_MOESM2_ESM.pdf]

```
a
AluSx    GGCCGGGCGCGGTGGCTCACGCCTGTAATCCCAGCACTTTGGGAGGCCGAGGCGGGCGGATCACCTGAGGTCAG
AluSp    ........................................................................G.
AluSz    ..........................................................................
AluSg    ................................................................--........
AluSq    ........................................................T.................
AluSc    ................................................................--.......A
AluY     ................................................................--........
AluYa5   ................................................................--........
AluYa8   ................................................................--........
AluYb8   ........................................................T......T--........
AluYb9   ........................................................T......T--........
AluYc1   ................................................................--........
b
11q14.1  ..T......T...........................................A....................

AluSx    GAGTTCGAGACCAGCCTGGCCAACATGGTGAAACCCCGTCTCTACTAAAAATAC-AAAAATTAGCCGGGCGTGG
AluSp    ..................A.........A.............................................
AluSz    ..........................................................................
AluSg    ..........................................................................
AluSq    ..........................................................................
AluSc    ...A.........T...................................................T........
AluY     ...A.........T......T....C............................A...................
AluYa5   ...A.........T..C...T..A.C............................A.................A.
AluYa8   ...A.........T..C...T..A.C........................C...-.....A...........A.
AluYb8   ...A.........T......T....A............................A................C..
AluYb9   ...A.........T......T....A............................A................C..
AluYc1   ...A.........T......T....C............................A...................
11q14.1  ..........-.......A....A..............................A...................

AluSx    TGGCGCGCGCCTGTAATCCCAGCTACTCGGGAGGCTGAGGCAGGAGAATCGCTTGAACCCGGGAGGCGGAGGTT
AluSp    ......AT..................................................................
AluSz    ..........................................................................
AluSg    ..........................................................................
AluSq    .....G....................................................................
AluSc    ...............G..........................................................
AluY     .....G.........G.................................G..G..................C..
AluYa5   .....G.........G...........T.....................G..G..................C..
AluYa8   .....G.........G...T.......T.....................G..G..................C..
AluYb8   .....G.........G.................................G..G...........A......C..
AluYb9   .....G.........G...........G.....................G..G...........A......C..
AluYc1   .A...G.........G.................................G..G..................C..
11q14.1  ...T.-.........G.................................G..A.......A..........C..

AluSx    GCAGTGAGCCGAGATCGCGCCACT-------GCACTCCAGCCTGGGCGAC-AGAGCGAGACTCCGTCTCA
AluSp    ..G...................T........................A..A.......A...........
AluSz    ......................................................................
AluSg    ......................................................................
AluSq    ...............................................A..A.......A...........
AluSc    ...........................................-..........................
AluY     ......................................................................
AluYa5   ................C.....................................................
AluYa8   ................C.....................................................
AluYb8   ...............T........GCAGTCC...G...G...............................
AluYb9   ...............T........GCAGTCC...G...G...............................
AluYc1   ......................................................................
11q14.1  .........A..A....T......................................A....G........
```
