# Supplementary material for: Insertion and deletion polymorphisms of the ancient AluS family in the human genome
Source: Mob DNA. 2017 Apr 24;8:6. doi: 10.1186/s13100-017-0089-9 (PMC5402677; doi:10.1186/s13100-017-0089-9)
Supplement: Supplementary file 5 — Functionally significant Alu sequence features annotated in TPRT insertion candidates (n = 7). (PDF 203 kb) [file 13100_2017_89_MOESM5_ESM.pdf]

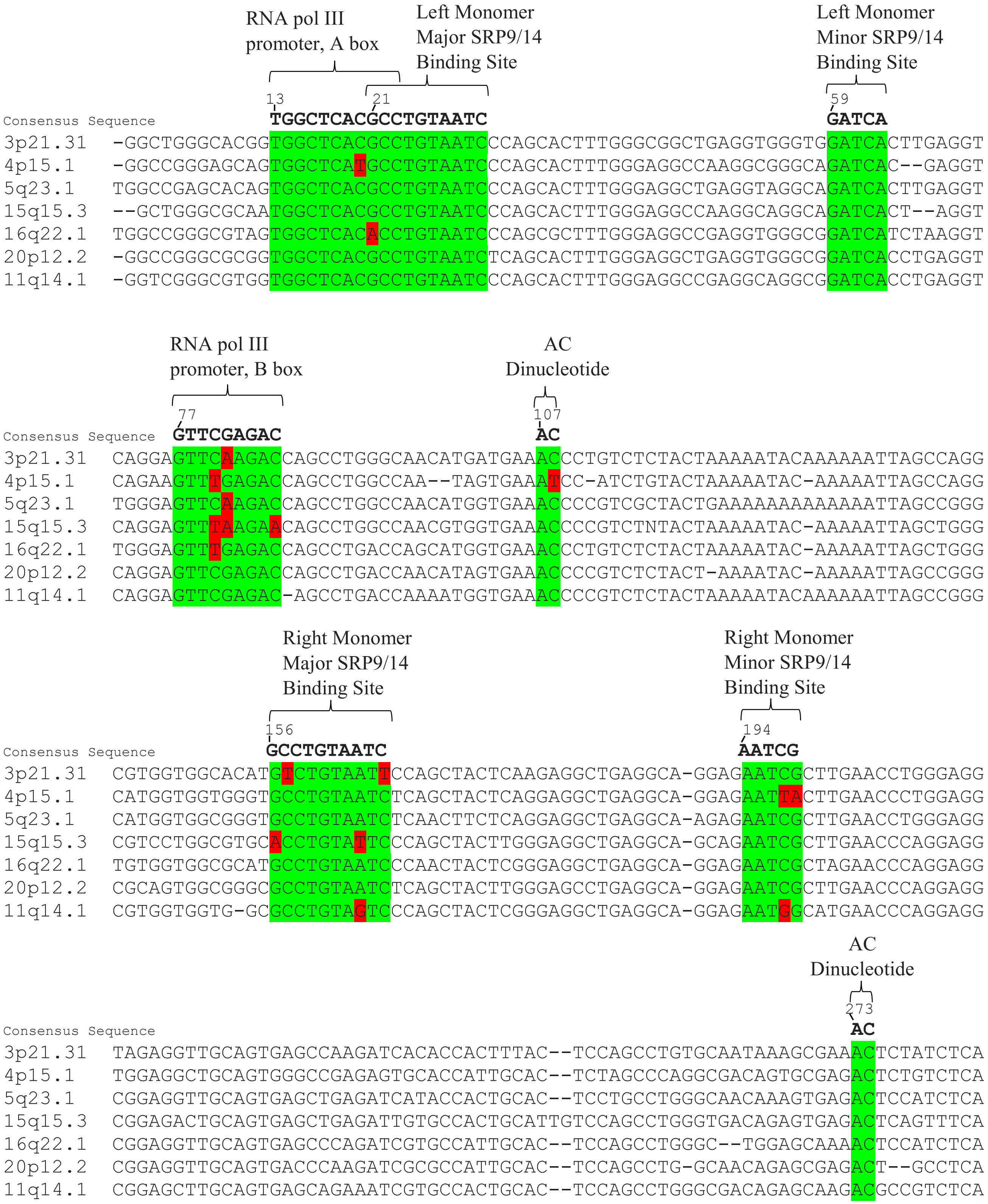

RNA pol III promoter, A box
Left Monomer Major SRP9/14 Binding Site
Left Monomer Minor SRP9/14 Binding Site
13
21
59
Consensus Sequence TGGCTCACGCCTGTAATC GATCA
3p21.31 -GGCTGGGCACGGTGGCTCACGCCTGTAATCCCAGCACTTTGGGCGGCTGAGGTGGGTGGATCACTTGAGGT
4p15.1 -GGCCGGGAGCAGTGGCTCATGCCTGTAATCCCAGCACTTTGGGAGGCCAAGGCGGGCAGATCAC--GAGGT
5q23.1 TGGCCGAGCACAGTGGCTCACGCCTGTAATCCCAGCACTTTGGGAGGCTGAGGTAGGCAGATCACTTGAGGT
15q15.3 --GCTGGGCGCAATGGCTCACGCCTGTAATCCCAGCACTTTGGGAGGCCAAGGCAGGCAGATCACT--AGGT
16q22.1 TGGCCGGGCGTAGTGGCTCACACCTGTAATCCCAGCGCTTTGGGAGGCCGAGGTGGGCGGATCATCTAAGGT
20p12.2 -GGCCGGGCGCGGTGGCTCACGCCTGTAATCTCAGCACTTTGGGAGGCTGAGGTGGGCGGATCACCTGAGGT
11q14.1 -GGTCGGGCGTGGTGGCTCACGCCTGTAATCCCAGCACTTTGGGAGGCCGAGGCAGGCGGATCACCTGAGGT
RNA pol III promoter, B box
AC Dinucleotide
77
107
Consensus Sequence GTTCGAGAC AC
3p21.31 CAGGAGTTCAAGACCAGCCTGGGCAACATGATGAAACCCTGTCTCTACTAAAAATACAAAAAATTAGCCAGG
4p15.1 CAGAAGTTTGAGACCAGCCTGGCCAA--TAGTGAAATCC-ATCTGTACTAAAAATAC-AAAAATTAGCCAGG
5q23.1 TGGGAGTTCAAGACCAGCCTGGCCAACATGGTGAAACCCCGTCGCTACTGAAAAAAAAAAAAATTAGCCGGG
15q15.3 CAGGAGTTTAAGAACAGCCTGGCCAACGTGGTGAAACCCCGTCTNTACTAAAAATAC-AAAAATTAGCTGGG
16q22.1 TGGGAGTTTGAGACCAGCCTGACCAGCATGGTGAAACCCTGTCTCTACTAAAAATAC-AAAAATTAGCTGGG
20p12.2 CAGGAGTTCGAGACCAGCCTGACCAACATAGTGAAACCCCGTCTCTACT-AAAATAC-AAAAATTAGCCGGG
11q14.1 CAGGAGTTCGAGAC-AGCCTGACCAAAATGGTGAAACCCCGTCTCTACTAAAAATACAAAAAATTAGCCGGG
Right Monomer Major SRP9/14 Binding Site
Right Monomer Minor SRP9/14 Binding Site
156
194
Consensus Sequence GCCTGTAATC AATCG
3p21.31 CGTGGTGGCACATGTCTGTAATTCCAGCTACTCAAGAGGCTGAGGCA-GGAGAATCGCTTGAACCTGGGAGG
4p15.1 CATGGTGGTGGGTGCCTGTAATCTCAGCTACTCAGGAGGCTGAGGCA-GGAGAATTACTTGAACCCTGGAGG
5q23.1 CATGGTGGCGGGTGCCTGTAATCTCAACTTCTCAGGAGGCTGAGGCA-AGAGAATCGCTTGAACCTGGGAGG
15q15.3 CGTCCTGGCGTGCACCTGTATTCCCAGCTACTTGGGAGGCTGAGGCA-GCAGAATCGCTTGAACCCAGGAGG
16q22.1 TGTGGTGGCGCATGCCTGTAATCCCAACTACTCGGGAGGCTGAGGCA-GGAGAATCGCTAGAACCCAGGAGG
20p12.2 CGCAGTGGCGGGCGCCTGTAATCTCAGCTACTTGGGAGCCTGAGGCA-GGAGAATCGCTTGAACCCAGGAGG
11q14.1 CGTGGTGGTG-GCGCCTGTAGTCCCAGCTACTCGGGAGGCTGAGGCA-GGAGAATGGCATGAACCCAGGAGG
AC Dinucleotide
273
Consensus Sequence AC
3p21.31 TAGAGGTTGCAGTGAGCCAAGATCACACCACTTTAC--TCCAGCCTGTGCAATAAAGCGAAACTCTATCTCA
4p15.1 TGGAGGCTGCAGTGGGCCGAGAGTGCACCATTGCAC--TCTAGCCCAGGCGACAGTGCGAGACTCTGTCTCA
5q23.1 CGGAGGTTGCAGTGAGCTGAGATCATACCACTGCAC--TCCTGCCTGGGCAACAAAGTGAGACTCCATCTCA
15q15.3 CGGAGACTGCAGTGAGCTGAGATTGTGCCACTGCATTGTCCAGCCTGGGTGACAGAGTGAGACTCAGTTTCA
16q22.1 CGGAGGTTGCAGTGAGCCCAGATCGTGCCATTGCAC--TCCAGCCTGGGC--TGGAGCAAAACTCCATCTCA
20p12.2 CGGAGGTTGCAGTGACCCAAGATCGCGCCATTGCAC--TCCAGCCTG-GCAACAGAGCGAGACT--GCCTCA
11q14.1 CGGAGCTTGCAGTGAGCAGAAATCGTGCCACTGCAC--TCCAGCCTGGGCGACAGAGCAAGACGCCGTCTCA
